# Adrenal mixed corticomedullary tumors: report of a case with molecular characterization and systematic review

**DOI:** 10.1007/s00428-025-04091-9

**Published:** 2025-04-02

**Authors:** Edurne Pérez-Béliz, Benjamín Alfonso Thorpe-Plaza, Everardo Josué Díaz-López, Lourdes Loidi, Carmen Villalba-Martín, Ihab Abdulkader-Nallib, José Manuel Cameselle-Teijeiro

**Affiliations:** 1 Department of Pathology, Clinical University Hospital of Santiago de Compostela, Galician Healthcare Service (SERGAS), Health Research Institute of Santiago de Compostela (IDIS), Travesía Choupana S/N, 15706 Santiago de Compostela, Spain; 2 Department of Surgery, Clinical University Hospital of Santiago de Compostela, Galician Healthcare Service (SERGAS), Health Research Institute of Santiago de Compostela (IDIS), Santiago de Compostela, Spain; 3 Division of Endocrinology and Nutrition, Center for Research in Molecular Medicine and Chronic Diseases (CIMUS), Clinical University Hospital of Santiago de Compostela, Galician Healthcare Service (SERGAS), UETeM, Health Research Institute of Santiago de Compostela (IDIS), University of Santiago de Compostela, Santiago de Compostela, Spain; 4 Galician Public Foundation for Xenomic Medicine (SERGAS-Xunta de Galicia), Santiago de Compostela, Spain; 5 Department of Radiology, Clinical University Hospital of Santiago de Compostela, Galician Healthcare Service (SERGAS), Health Research Institute of Santiago de Compostela (IDIS), Santiago de Compostela, Spain; 6 University of Santiago de Compostela, Santiago de Compostela, Spain

**Keywords:** Mixed corticomedullary tumor, Adrenal tumor, *GNAS*, *AKAP13*, *EPAS1*, Arterial hypertension

## Abstract

Adrenal mixed corticomedullary tumors (MCMTs) are rare lesions showing a mixture of two cell populations of cortical and medullary lineage. We describe an MCMT case presented in a 56-year-old woman with a history of arterial hypertension and high levels of aldosterone, accompanied by a review of the literature. The adrenalectomy specimen showed a well-circumscribed nodule of 30 mm in size, containing 60% of cells with a cortical phenotype (positive for α-inhibin and melan-A) and 40% of cells with a medullary phenotype (positive for chromogranin-A, GATA-3 and somatostatin receptor 2). There was no significant mitotic activity, necrosis, nor lymphovascular invasion. The *GNAS* p.(Arg844Cys) mutation, as well as variants of uncertain significance *AKAP13* p.(His641Pro) and *EPAS1* p.(Ser478del) were detected in the tumor. MCMT is more common in women (75%) with a mean age of 46.6 years (range 16–78). Most patients present with hypertension (79%), frequently associated with Cushing’s syndrome, (39%), diabetes (21%), aldosteronism (15%), and/or hyperandrogenism (6%). Laboratory data showed elevated levels of both cortisol and cathecholamines and/or their metabolites in more than 50% of cases, supporting the dual nature of the tumor. Most MCMTs are benign, but aggressive behavior was detected in four (12%) cases, all of them showing large size (80–220 mm), poor delimitation, venous invasion, necrosis, and/or high proliferation rates. The pathogenesis is unknown, but our findings suggest a tumor histogenesis from the cortical cellular component through the regulation of the protein kinase A pathway and secondary proliferation of the medullary component.

## Introduction

Mixed corticomedullary tumors (MCMTs) of the adrenal gland are very rare tumors first described by Mathison and Waterhouse in 1969 [1]. This type of neoplasm displays an intermingled proliferation of both cortical and medullary cells of the adrenal gland, a surprising fact given that steroid-producing cortical cells derive from the mesoderm while catecholamine-producing medulla cells derive from the neural crest [2]. The clinical presentation of MCMT may vary in relation to the secretion of adrenal hormones such as catecholamines, adrenal steroid hormones, and/or ectopic production of adrenocorticotropic hormone (ACTH) [1, 3–31]. Thus, the preoperative diagnosis is usually adrenal cortical adenoma or pheochromocytoma, based on imaging and hormonal analysis, but histopathological evaluation together with immunohistochemical study is mandatory for the definitive diagnosis due to the dual nature of the tumor. Clinicians should be aware of MCMT to avoid a possible hypertensive crisis during surgery. To our knowledge, only 32 cases of MCMT have been described to date [1, 3–31]. Due to its rarity and the different embryonic lineage of the two tumor cellular components, the clinicopathological features and pathogenesis of MCMT are not well-known. Here, we report a new case of MCMT with molecular study, along with a systematic review of the literature to clarify the main characteristics of this rare tumor.

## Materials and methods

### Case

**Fig. 1:**
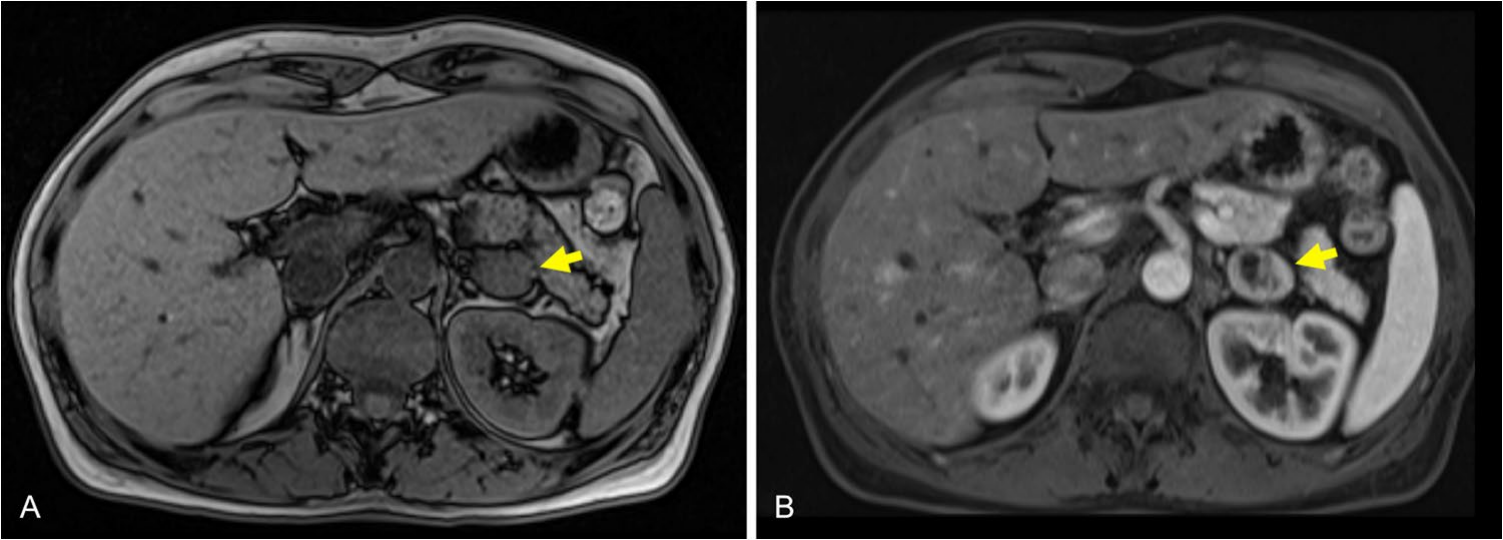
Magnetic resonance images demonstrate a left adrenal mass (arrows) with solid and cystic components. The solid component shows no significant loss of signal intensity in T1-w out-of phase sequence, which meanss that the lesion does not contain a significant amount of intracellular lipids (**A**). The arterial phase demonstrates dishomogeneous enhancement of the lesion and a lack of enhancement of the cystic component, a finding more frequently observed in pheocromocytoma (**B**). The diferential diagnosis includes lipid poor adenoma and pheocromocytoma

A 56-year-old woman, with a 5-year history of uncontrolled hypertension treated with a calcium channel blocker, was referred to our hospital due to muscle cramps and chronic spontaneous hypokalemia despite treatment with oral potassium. Her blood pressure was 150/90 mmHg. Laboratory tests showed a serum potassium level of 3.1 mmol/L. With the clinical suspicion of primary aldosteronism (PA), the patient underwent a hormonal analysis, which showed high levels of plasma aldosterone (325.0 pg/mL [normal upper limit: 160 pg/mL]) and low plasma renin activity (0.2 ng/mL/h) with an aldosterone-to-renin ratio (ARR) of 163.5 ng/dL. She then underwent a captopril challenge test which did not show a suppressed level of plasma aldosterone concentration confirming a PA diagnosis. The urinary catecholamines were normal. Magnetic resonance imaging studies showed a mixed solid-cystic lesion measuring 26 mm in the left adrenal gland. The solid component showed no signal fall in T1 opposed-phase versus T1 in phase, and the imaging differential diagnosis included pheochromocytoma and adrenal adenoma poor in lipids (Fig. 1). A left posterior retroperitoneoscopic adrenalectomy was performed without complications. Forty-two months after the intervention, poor blood pressure control persisted, requiring three antihypertensive drugs (angiotensin receptor, thiazide diuretic, and alpha blocker).

#### Pathological and immunohistochemical analysis

The surgical specimen was fixed in neutral, phosphate-buffered, 10% formalin and totally included in paraffin blocks. Paraffin-embedded sections were stained with hematoxylin and eosin; immunohistochemical studies were also performed on 4-μm-thick paraffin sections using a peroxidase-conjugated-labeled dextran polymer (Dako EnVision Peroxidase/DAB; Dako, Glostrup, Denmark), with 3,3′-diaminobenzidine as the chromogen and a series of primary antibodies as follows: α-inhibin (clone: R1, concentration: ready-to-use, antigenic recovery treatment: pH 9, manufacturer: DAKO, Glostrup, Denmark), Melan-A (A103, ready-to-use, pH 9, DAKO), chromogranin-A (DAK-A3, 1:100, pH 6, DAKO), GATA3 (L50-823, 1:50, pH 9, Gennova, Sevilla, Spain), somatostatin receptor 2 (SSTR2) (polyclonal, rabbit, 1:50, pH 9, Gennova), synaptophysin (DAK-SYNAP, ready-to-use, pH 9, DAKO), SDHB (ZR339, 1:200, pH 9, Zeta, Madrid, Spain), S100 (polyclonal, rabbit, ready-to-use, pH 9, DAKO), p53 (DO-7, ready-to-use, pH 9, DAKO), OCT3/4 (ready-to-use, pH9, DAKO), SALL4 (AP10477C 1:50, pH9, Gennova), and Ki67 (MIB1, ready-to-use, pH6, DAKO).

#### Genetic analysis

DNA extraction from a formalin-fixed paraffin-embedded tumor tissue sample, containing both cell types, was undertaken using the GeneRead DNA FFPE kit (Qiagen, Germany). Next-generation sequencing (Illumina NovaSeq 6000) was made of the entire coding region as well as the flanking intronic regions of the genes included in the exome. Capturing the regions of interest was performed using KAPA HyperExome V2 Probes (Roche, CA, USA). The data analysis was carried out using computer tools: DRAGEN-OS 0.2020.0819, SAMBLASTER 0.1.26, GATK v4.4.0, Pindel 0.2.5b9, Picard 3.0.0, mosdepth 0.3.3, bedtools v2.31.0, samtools 1.17, ExomeDepth 1.1.16, Halogrep v2.4.0, SnpEff, ANNOVAR. A panel of genes commonly altered in adrenal tumors was analyzed, including *ACO1*, *AKAP13*, *ATRX*, *CSDE1*, *CTNNB1*, *EGLN1*, *EGLN2*, *EPAS1*, *FH, GNAS*, *HRAS*, *IDH1*, *IDH2*, *KF1B*, *MAML3*, *MAX*, *MDH2*, *MEN1*, *NF1*, *PDE8A*, *PRKACA*, *PRKACB*, *PRKAR1A*, *RET*, *SDHA*, *SDHB*, *SDHC*, *SDHD*, *TMEM127*, and *VHL*. Median coverage was 194 × . Genetic variants were reported according to the Human Genome Variation Society, and the classification of pathogenicity was performed in accordance with the standards of pathogenicity of somatic variants in cancer (32).

### Literature review

#### Study selection

All articles, including single cases or case series with a MCMT diagnosis up until December 31, 2024, were investigated from the electronic databases Medline, PubMed, and Google Scholar using the words “corticomedullary,” “mixed,” and “adrenal” as a specific search strategy. No additional contact was made with the articles’ authors.

#### Data abstraction and analysis

From each publication, we investigate the following information: age, sex, clinical features, surgical treatment, tumor size, hormone production, immunohistochemistry, proliferative index, clinical behavior, molecular alterations, and concurrent pathology. We also perform a comparative analysis of the data.

## Results

### Case

#### Pathological and immunohistochemical findings

**Fig. 2:**
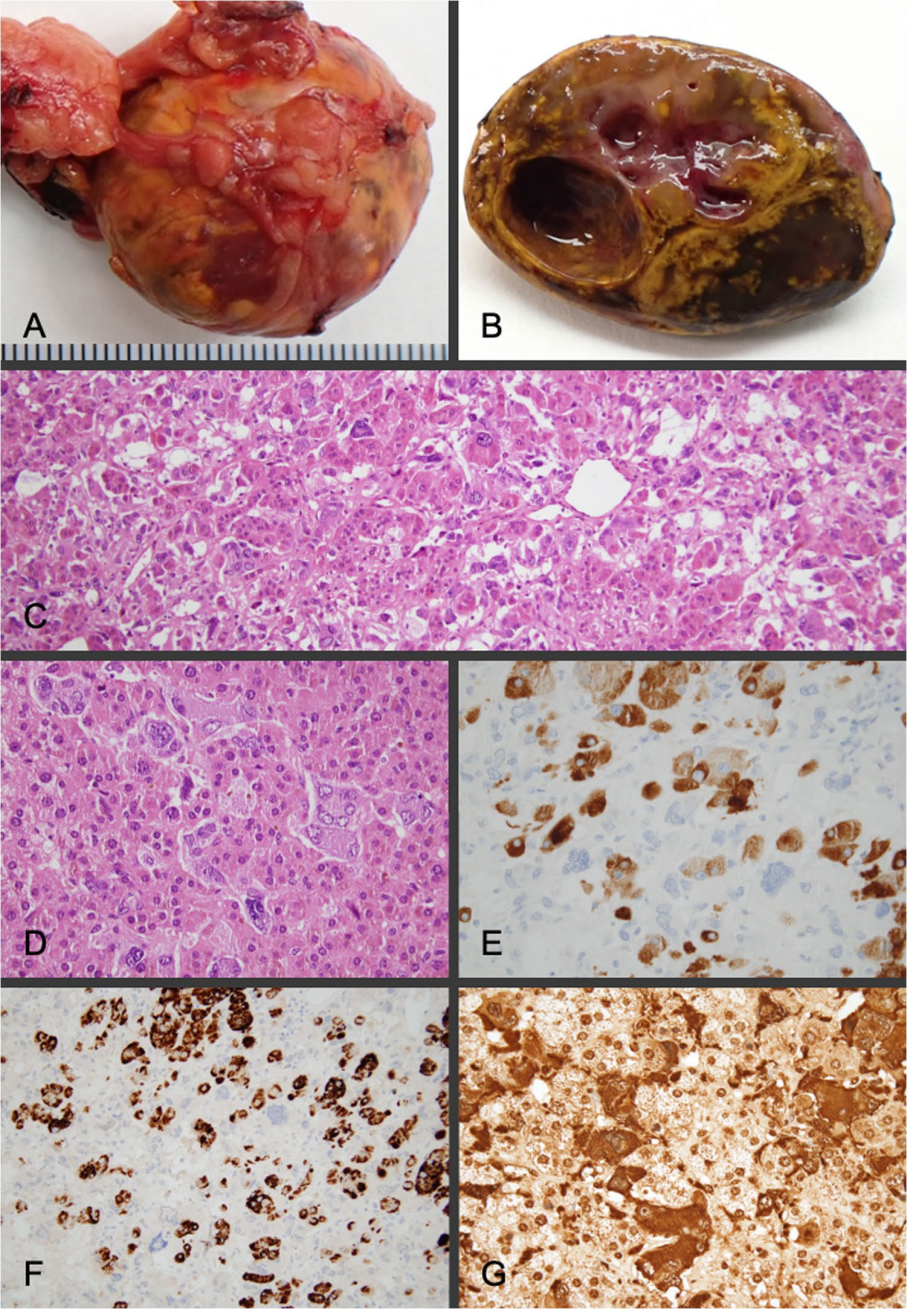
Mixed corticomedullary tumor. External appearance of the resection specimen (**A**) whose section (**B**) showed a yellowish tumor with interspersed pink and brownish areas and cystification. Microscopically, a combination of cells with eosinophilic cytoplasm with a central nucleus (cortical adenoma type) was observed, along with another tumor cell component displaying granular basophilic to amphophilic cytoplasm (pheochromocytoma type) (**C** and **D**). Tumor cells with a cortical phenotype were positive for inhibin (**E**) and melan-A (**F**), while those with a chromaffin phenotype stained for chromogranin (**G**)

The adrenalectomy specimen showed a well-circumscribed nodule measuring 30 × 28 × 20 mm. On section, the tumor was yellowish with some pink areas and two cystic formations of 3 and 8 mm in diameter (Fig. 2). Histologically, an intimate admixture of two different tumor cell populations was observed in the nodule. Some tumor cells showed large, eosinophilic, and clear cytoplasms with a small central nucleus, which resembled cortical adenoma cells, while the other tumor cell component showed cells displaying granular basophilic to amphophilic cytoplasm and a vesicular nucleus similar to normal chromaffin cells (Fig. 2). Nuclear atypia, multinucleation, and cellular pleomorphism were occasionally seen in this second cellular type. There was no significant mitotic activity (less than 1 mitotic figure per 2 mm^2^), no necrosis, no lymphovascular invasion, nor areas of tumor infiltration detected anywhere in the tumor. A thin rim of compressed adrenal cortical tissue was identified at the periphery. About 60% of tumor cells (cortical adenoma component) were immunoreactive for α-inhibin and Melan-A, while 40% of the other tumor cells (pheochromocytoma component) were positive for chromogranin-A, GATA3, and somatostatin receptor 2 (Fig. 2). Positivity for synaptophysin and SDHB was detected in both cellular components. S100-positive sustentacular cells were identified scattered throughout the tumor. The expression pattern for p53 was wild-type. No reactivity for OCT3/4 or for SALL4 was detected in tumor cells. The Ki67 index was 1.5%. A diagnosis of mixed corticomedullary tumor of the left adrenal gland was made.

#### Genetic findings

In the present analysis, the mutation c.2530C > T, p.(Arg844Cys) of the *GNAS* gene (NM_080425.4) was detected with a variant allele frequency (VAF) of 17.8%. Variant c.1922A > C, (p.His641Pro), (VAF 8.6%), detected in *AKAP13* (NM_006738.6), as well as variant c.1432_1434delAGC, p.(Ser478del) (VAF 1.4%) detected in *EPAS1* (NM_001430.5), were considered to be of uncertain significance.

### Review findings

In this review, we have identified 30 articles [1, 3–31, 33, 34] describing 32 cases of MCMT, with the new patient described in this article representing case number 33. These tumors are more common in women (75%) with a mean age of 46.63 years (median 48, range 16–78). Clinically, the majority of patients presented with hypertension (78.87%) [1, 4–8, 10–13, 16–18, 21, 23–26, 28, 30, 31], and abdominal pain [8, 19, 20, 22, 29], incidental finding [14], or Takotsubo syndrome [27] were other presentation forms of MCMT. Concomitant endocrinological diseases were Cushing’s syndrome or subclinical Cushing’s in 13 cases (39.39%) [1, 5, 6, 8, 9, 11, 14, 21, 23, 25, 26, 28], diabetes in seven cases (21.21%) [4, 6, 7, 11, 12, 18, 23], and PA in five (15.15%) cases (including our case) (6,7,28); of these patients with PA, four cases had overt PA (elevated plasma aldosterone, suppressed renin and spontaneous hypokalemia) (6,7,28). Hiperandrogenism was detected in two (6%) women [3, 13].

Laboratory investigations evidenced tumor production of cortisol in 22 (66.66%) cases [1, 4–6, 8–14, 20–26, 28–30], and elevation of cathecholamines (epinephrine and/or norepinephrine) and/or their metabolites in 20 (60.6%) cases [1, 4–6, 11, 12, 15–19, 21, 23, 24, 26–28, 30, 31]. Increased levels of testosterone or dehyroepiandrosterone were noted in four (12.5%) cases [3, 13, 17, 25] fitting with the functional capacity of the tumor cells of the adrenal cortex. All these hormonal alterations explain the variety of clinical manifestations of MCMT and reflect the dual nature of the tumor. Aggressive behavior with liver metastases has been reported in four (12.12%) cases [6, 17, 20, 22].

Radiologically, MCMT usually appears as a well-delimited solid mass with a mean diameter of 62.78 mm (median 45, range 24–220) not showing preference for either adrenal gland [1, 3–31]. Including the tumor here described, the presence of cysts within the tumor was only reported in three (9%) cases [20, 31], with one having malignant behavior [20].

The main positive markers for adrenocortical and medullary neoplasms, including pheochromocytoma and/or ganglioneuroma reported in MCMT, are summarized in Table 1. By electron microscopy, it was observed that cells with cytoplasmic dense-core granules characteristic of adrenal medullary cells alternated with a second cell type of adrenal cortical origin that contained abundant lipid inclusions and smooth endoplasmic reticuli without dense-core secretory granules [4, 6, 9, 10]. Isolated cases of MCMT coexisting with adipose tissue [13], myelolipoma [9, 15], and spindle cell sarcoma [6] have also been reported. Concomitant clear cell renal cell carcinoma [18] and liver angiomatosis [19] were reported in two MCMT patients.

**Table 1:** Immunohistochemical and ultrastructural markers of MCMT and follow-up

References	Positive immunohistochemical markers and ultrastructural studies	Mitotic index	Ki-67 (%)	Follow-up (months)
Mathison et al.^1^	ND	ND	ND	NET (11)
Takahashi et al.^3^	ND	ND	ND	NET (6)
Akai et al.^4^	ND, EM*	ND	ND	ND
Ohta et al.^5^	CHR, NPY, PYY, SMT, CT, NSE, NT	ND	ND	NET (10)
Michal et al.^6^	CHR, SYN, EMA, EM*	Low^§^	ND	NET (24)
	CHR, SYN, EMA, CT, EM*	Low	ND	NET (72)
Delèvaux et al.^7^	NSE	ND	ND	ND
Wieneke et al.^8^	CHR, SYN, S100^†^, INH	0	ND	NET (4)
	CHR, SYN, S100^†^, INH	0	ND	NET (360)
Chu et al.^9^	CHR, SYN, INH, mel-A, CALR, EM*	0	ND	ND
Ma et al.^10^	CHR, SYN, INH, mel-A, EM*	ND	ND	ND
Lee et al.^11^	CHR, SYN, INH, mel-A	ND	ND	ND
Kimura et al.^12^	CHR, p450c21	ND	ND	ND
Trimeche et al.^13^	CHR	ND	ND	ND
Alexandraki et al.^14^	CHR, SYN	0	ND	NET (36)
Singh et al.^15^	CHR, SYN, S100^†^, INH	0	ND	ND
Lau et al.^16^	CHR, SYN, S100^†^, INH, mel-A	0		NET (8)
Turk et al.^17^	CHR, SYN, S100^†^, INH, mel-A	Brisk^‡^	45	Recurrence and liver metastasis (4)^¶^
Kaneko et al.^18^	CHR, SYN	ND	ND	ND
Donatini et al.^19^	CHR, SYN, S100^†^, INH, mel-A	2	ND	NET (4)
Michalopoulos et al.^20^	CHR, SYN, S100^†^, NSE CAL and CKAE1/AE3 (focally positive)	< 5	5	Died with liver and lung metastases (18)^¶^
Lwin et al.^21^	CHR, SYN, S100^†^, INH, mel-A	0	< 1	NET (34)
Alsabek et al.^22^	CHR, SYN, INH, CALR	8	ND	Alive with liver metastases (20)^¶^
Duan et al.^23^	CHR, SF1 ALDH1, CD44, CD133, nestin, NGFR and SOX9 (positive in scattered cells)	ND	ND	NET (18)
Kanzawa et al.^24^	CHR, SYN, INH, SF1, INSM1, TH, DBH, PNMT, CYP11β1	ND	< 1	NET (36)
Ramírez et al.^25^	CHR, SYN, S100^†^, INH, SF1, CALR, CD56	6	40	Alive with liver metastases (16)^¶^
Chiou et al.^26^	CHR, INH, CD34, SOX2, OCT4	ND	2	NET 84)
Inoue et al.^27^	CHR, SF1, CYP11β1, 3β-HSD, p450c21	ND	ND	ND
Yoshida et al.^28^	CHR, SF1, SYN, TH, CYP11β1, ACTH	ND	ND	NET (108)
Engur et al.^29^	ND	ND	2.1	NET (3)
Patel et al.^30^	CHR, SYN, INH, SDHB	0	ND	NET (1)
Maradana et al^31^	CHR, INH, SF1, MART-1	ND	ND	NET(30)
Present case	CHR, SYN, INH, mel-A, GATA3, SSTR2, SDHB	< 1	1.5	NET (42)

*ND* not done, *NET* no evidence of tumor, *CHR* chromogranin, *NPY* neuropeptide Y, *PYY* peptide YY, *SMT* somatostatin, *CT* calcitonin, *NSE* neuron-specific enolase, *NT* neurotensin, *SYN* synaptophysin, *EMA* epithelial membrane antigen, *INH* α-inhibin, *CALR* calretinin, *mel*-*A*, melan A, *CKAE1/AE3* pan-cytokeratin cocktail, *SF1* steroidogenic factor 1, *ALDH1* aldehyde dehydrogenase 1, *NGFR* nerve growth factor receptor, *SOX9* sex determining region Y-box 9, *INSM1* insulinoma-associated protein 1, *TH* tyrosine hydroxylase, *DBH* dopamine beta hydroxylase, *PNMT* phenylthanolamine-N-methyl tranferase, *CYP11β1* cytochrome P450 11beta1, *SOX2* sex determining region Y-box 2, *OCT4* POU class 5 homeobox 1, *3β-HSD* 3beta-hydroxysteroid dehydrogenase, *p450c21* cytochrome P450 oxidase, *MART-1* melanoma antigen recognized by T cells

^*^Electron microscopy (EM) study demonstrated both cells with ultrastructural characteristics of adrenocortical cells and adrenomedullary cells

^§^A third tumor component of spindle cell sarcoma with numerous mitoses was found in this case

^†^S100 positive sustentacular cells

^‡^Brisk mitotic activity with atypical mitotic figures

^¶^The primary tumor showed large size, frequent mitoses with atypical forms, nuclear pleomorphism, tumor necrosis, vascular invasion, and/or infiltration of the surrounding fat

## Discussion

Mixed corticomedullary tumors (MCMTs) of the adrenal gland are uncommon neoplasms containing an intimate mixture of adrenal cortical adenoma and medullary cells [1, 3–31, 33, 34]. These are usually cortical adenoma cells and pheochromocytoma cells but less commonly cortical adenoma cells with both ganglioneuroma cells and pheochromocytoma cells (16,30), or even more rarely a combination of cortical adenoma and ganglioneuroma cells [3]. While in one case of MCMT, the cortical and medullary tumor cells were arranged in two distinct layers [23]; these tumors are characteristically composed of an admixture of adrenocortical adenoma cells and medullary cells [1, 3–22, 24–31]. The dual nature of MCMT has been confirmed both ultrastructurally [4, 6, 9, 10] and immunohistochemically (Table 1). True MCMT must be differentiated from the so-called collision tumors in which two simultaneous independent (well-delimited) tumor masses of different lineage are identified. For this reason, cases of simultaneous ganglioneuroma and cortical adenoma [35], as well as cases of compound adrenal medullary tumors (pheochromocytoma and ganglioneuroma) with ipsilateral [36] or contralateral cortical adenoma [37], are not true MCMT. Although some cases of MCMT have been reported in patients with concomitant clear cell renal cell carcinoma [18] and liver angiomatosis [19], there is no clear pathogenic relationship. Most MCMTs are benign and simple excision is the treatment of choice. The only four malignant tumors published were large (measuring 80–220 mm in diameter) and displayed histopathological features of aggressiveness such as poor delimitation, venous invasion, and/or necrosis. In three of these four malignant cases, the proliferation index was high with numerous mitoses and/or Ki-67 ≥ 40% [6, 17, 22].

The histogenesis of MCMT is controversial. In our opinion, it seems reasonable to exclude collision tumors [6, 9, 28] because of the intimate admixture of both cortical and medullary tumor components in true MCMTs. True MCMT could arise from alterations in the interaction between cortical and chromaffin cells [16, 28], including paracrine secretion of growth factors [13] with hyperplastic and/or neoplastic changes in cortical cells and secondary proliferation of chromocytes via phenylethanolamine-N-methyltransferase [7, 11, 23]. Both a single stem cell [11] and a cancer stem cell [23] histogenesis have also been proposed; in fact, and in a few MCMTs, some cells with double immunohistochemical positivity for insulinoma-associated protein 1 and alpha-inhibin [24] or for chromogranin with alpha-inhibin [25] or with steroidogenic factor 1 [27] have been detected. A case of MCMT has been reported in the context of neurofibromatosis type 1 [11] and another case was associated with the germline *FGFR4*-G388R variant [24]. More recently, it has been proposed that the combination of germline mutations involved in the regulation of stemness and cell proliferation together with somatic mutations related to glycolysis and the citrate cycle could contribute to the development of MCMT [26]. In our case, a somatic *GNAS* gene mutation was identified; this is an alteration typically associated with cortisol-producing adrenal cortical adenomas and McCune Albright syndrome (a somatic *GNAS* mosaicism) [38, 39] but not related to adrenal medullary tumors. Thus, our findings support an MCMT origin stemming from the proliferation of the cortical cellular component through the regulation of the protein kinase A (PKA) pathway [40], with secondary reactive proliferation of the medullary component. And although of uncertain significance, the additional detection of the *AKAP13* gene variant, which is involved in encoding proteins that bind to the PKA regulatory subunit [41], highlights the relevance of this signaling pathway in MCMT. The other variant of uncertain significance was detected in the *EPAS1* gene, which is involved in signal transduction pathways related to oxygen levels and associated with rare cases of paragangliomas or pheochromocytomas with hemoglobin disorders [42, 43].

We can conclude that adrenal gland MCMTs are very rare lesions showing a mixture of two cell populations of cortical and medullary lineage. MCMT is more common in women with a wide range in age at presentation. Most patients present with hypertension, frequently coexisting with Cushing’s syndrome, diabetes, aldosteronism, and/or hyperandrogenism. In more than 50% of cases, the patients show elevated levels of both cortisol and cathecholamines and/or their metabolites, supporting the dual nature of the tumor. Most MCMTs are benign, but malignancy was detected in four (12%) cases, all of them showing large size, poor delimitation, venous invasion, necrosis, and/or high proliferation rates. Although the pathogenesis has not been established and the data are limited, our findings suggest a tumor histogenesis from the cortical cellular component through the regulation of the protein kinase A pathway with secondary proliferation of the medullary component.

## Data Availability

All data generated or analyzed during this study are included in this published article.
